# Estimating variability in grain legume yields across Europe and the Americas

**DOI:** 10.1038/srep11171

**Published:** 2015-06-08

**Authors:** Charles Cernay, Tamara Ben-Ari, Elise Pelzer, Jean-Marc Meynard, David Makowski

**Affiliations:** 1 INRA, UMR 211 Agronomie, F–78850, Thiverval–Grignon, France; 2 AgroParisTech, UMR 211 Agronomie, F–78850, Thiverval–Grignon, France; 3 INRA, UMR 1018 Sciences pour l’Action et le Développement: Activités, Produits, Territoires, F–78850, Thiverval–Grignon, France; 4 AgroParisTech, UMR 1018 Sciences pour l’Action et le Développement: Activités, Produits, Territoires, F–78850, Thiverval–Grignon, France

## Abstract

Grain legume production in Europe has recently come under scrutiny. Although legume crops are often promoted to provide environmental services, European farmers tend to turn to non-legume crops. It is assumed that high variability in legume yields explains this aversion, but so far this hypothesis has not been tested. Here, we estimate the variability of major grain legume and non-legume yields in Europe and the Americas from yield time series over 1961–2013. Results show that grain legume yields are significantly more variable than non-legume yields in Europe. These differences are smaller in the Americas. Our results are robust at the level of the statistical methods. In all regions, crops with high yield variability are allocated to less than 1% of cultivated areas. Although the expansion of grain legumes in Europe may be hindered by high yield variability, some species display risk levels compatible with the development of specialized supply chains.

The European Union (EU) recently stressed the importance of increasing its domestic production of grain legume crops^1^. Two reasons were outlined; the first is the necessity to reduce EU’s dependency on soybean imports from the Americas, and the second to reduce negative environmental impacts associated with intensive cereal production. The EU’s deficit in grain legume production takes its roots in the General Agreement on Tariffs and Trade in 1947 followed by the Blair House Agreement in 1992. These agreements allowed non-taxable protein imports from the Americas. This resulted in a competitive disadvantage for the EU’s grain legume production. Since 1974, grain legume cultivated areas sporadically increased following public support measures (i.e., price supports and subsidies from the Common Agricultural Policy). Legumes cultivated areas represented 1.8% of total European agricultural area in 2013 (compared to 14.5% in North America and 25.5% in South America in the same year)^2^. This is despite grain legumes representing a significant source of protein^3^,^4^,^5^,^6^ and a mean to reduce the reliance of arable cropping systems on synthetic nitrogen fertilizer and pesticides^7^,^8^,^9^,^10^.

Producing grain legumes is often viewed as risky by European farmers, who tend to prefer cultivating non-legume species such as cereals, oilseeds and tubers^11^,^12^,^13^,^14^. Several authors have in fact hypothesized that a large adoption of legume crops by European farmers is hampered by frequent losses due to high inter-annual yield variability^14^,^15^,^16^. Still, thus far, grain legume yield variability and risks of yield loss have never been quantitatively analyzed. A rigorous analysis of such risks is crucial for decision makers to define and promote efficient agro-environmental policies for grain legumes in Europe. An accurate estimation of yield loss distribution is also a key element for developing insurance systems against yield loss^17^,^18^,^19^,^20^. To date, only qualitative information from either country-level surveys or expertize is available on this topic^12^,^13^,^14^,^15^,^16^,^21^.

In this study, we quantitatively analyzed inter-annual yield variability and risks of yield loss for the major legume and non-legume yields at the scale of large world regions over 1961–2013. Historical time series were used to compute yield anomalies - defined as normalized yield residuals - for four European and two American regions. Three different risk measures were then estimated from yield anomaly distributions for each species in each region: (i) variance of yield anomalies, (ii) 10^th^ percentile of yield anomalies and (iii) expected yield loss. We compared each of these three measures applied to legumes and to non-legumes and classified crops along a risk gradient. We then investigated whether species characterized by higher levels of variability tend to be allocated to smaller proportions of total cultivated areas. Our results reveal that in Europe, the yields of legume crops are generally more variable than non-legumes but that levels of variability vary strongly both between crops and European sub-regions. Importantly, differences between legumes and non-legumes are much smaller in the Americas. These results are robust at the level of the statistical methods used to calculate yield anomalies (polynomial regression or local regression). This study also shows that species characterized by high levels of yield variability are allocated to less than 1% of total cultivated areas (i.e., legumes plus non-legumes). Production of grain legumes in Europe is thus likely to be hindered by high levels of yield variability occurring in this world region. Our analysis however provides a contribution for targeting low risk grain legume species in the prospect of expanding plant protein production in Europe.

## Results

### Grain legume yields are more variable than non-legume yields in Europe

**Figure 1:**
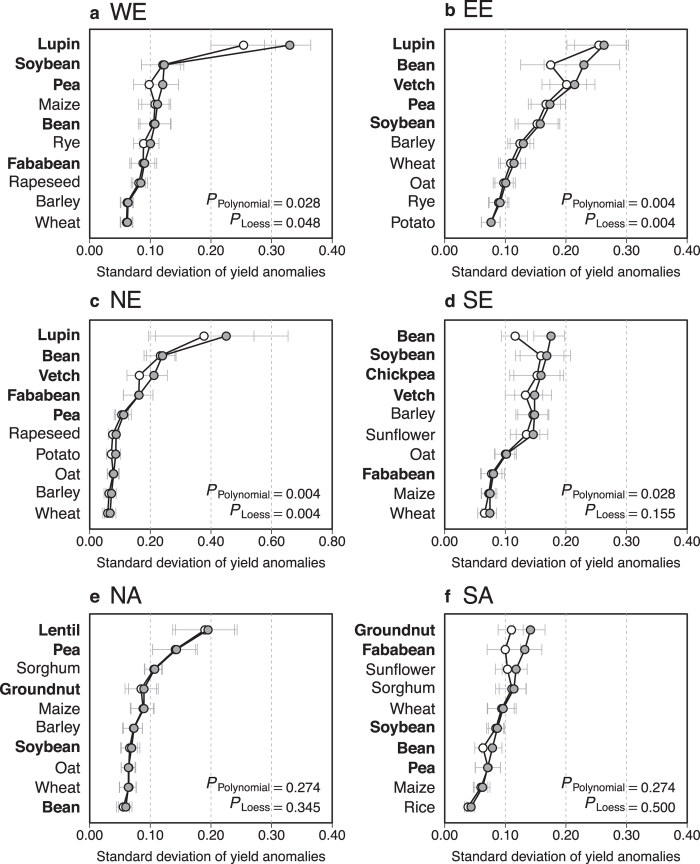
Standard deviation of yield anomalies for 10 crops in Europe and the Americas over 1961-2013. Standard deviation of yield anomalies for 10 crops in four European regions (i.e., Western Europe (WE), Eastern Europe (EE), Northern Europe (NE) and Southern Europe (SE)) and two American regions (i.e., North America (NA) and South America (SA)) over 1961-2013. Polynomial (grey points) and local ‘loess’ (empty points) regression models are used to calculate yield anomalies (relative differences between yield data and yield trend). Horizontal lines correspond to 95% confidence intervals estimated by bootstrap (10,000 samples). Among the 10 crops, 5 are legume crops (bold names) and 5 are non-legume crops (non-bold). All crops are ranked according to standard deviation of yield anomalies calculated using the polynomial model (decreasing order). *P*_Polynomial_ and *P*_Loess_ correspond to the p-value of the Wilcoxon rank test on a hypothesis assuming that ranks of legume and non-legume crops do not differ (against the alternative that legume crop ranking is lower) and are computed using polynomial and loess regression respectively. The number of yield data includes 53 observations for most crops and regions. There are two exceptions: soybean yield data in Western Europe includes 41 observations and lupin yield data in Northern Europe includes 22 observations. Grey vertical dashed lines represent the standard deviation values of 0.10, 0.20 and 0.30 in all regions but 0.20, 0.40 and 0.60 in Northern Europe.

**Figure 2:**
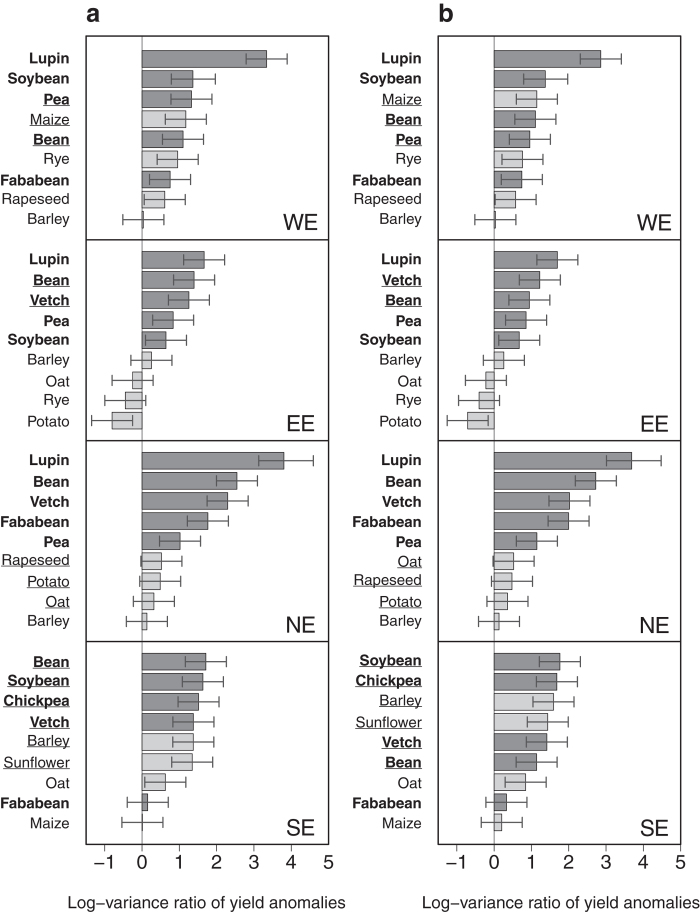
Log-variance ratio of yield anomalies for 9 crops compared to wheat in four European regions. Variances are calculated over 1961-2013 and log-transformed in Western Europe (WE), Eastern Europe (EE), Northern Europe (NE) and Southern Europe (SE). Yield anomalies are calculated using both regression models (polynomial (**a**), loess (**b**)). Horizontal lines correspond to 95% confidence intervals. Among the 9 crops, 5 are legume crops (bold names and dark grey bars) and 4 are non-legume crops (non-bold names and light grey bars). All crops are ranked according to variance ratio values (decreasing order). Crops that change in ranking between the polynomial and loess regression models used are underlined.

We analyze yield anomaly distributions for the five most cropped legume and non-legume species for three complementary risk measures in four European regions (see Methods section). A classification of standard deviation of yield anomalies shows that, in Europe, the majority of the five most cropped grain legumes are characterized by higher standard deviation values than the five most cropped non-legume crops (Fig. 1a–d). In all four European regions (i.e., Western, Eastern, Northern and Southern Europe), the three most variable crop yields are grain legumes and, in Eastern and Northern Europe, all five selected legumes have the highest standard deviations in yield. In Southern Europe, four out of five of the most variable crops are grain legumes. Ranks of legume species are overall significantly higher (indicating higher standard deviations) than non-legume ranks in all considered regions (*P* < 0.05), with one exception (Southern Europe with yield anomalies calculated using the local regression method, Fig. 1d). Compared to wheat (the 1^st^ European crop in terms of total cultivated area), variance of yield anomalies is systematically and significantly higher for legume species in all European regions, with only one exception (fababean in Southern Europe) (Fig. 2a). In particular, lupin has the highest variance ratio (see Methods section) for all three European regions where it is among the five most cropped legumes (i.e., in Northern, Eastern, and Western Europe); variance of yield anomalies of lupin is 5 to 45 times higher than that of wheat. Bean ranks first in Southern Europe and soybean is the second most variable crop in both Western and Southern Europe. Compared to wheat, variance of soybean yield anomalies is roughly four times higher in Western Europe (almost two times and above five times higher in Eastern and Southern Europe, respectively). Wheat shows the lowest yield standard deviation for all legume and non-legume crops studied in all European regions except Eastern Europe where potato is the least variable crop. Among legume species, the least variable specie is obtained for fababean in Southern and Western Europe, and for soybean and pea in Eastern and Northern Europe respectively. The second risk measure (10^th^ percentile of yield anomaly distributions) gives a somewhat equivalent ranking for studied species, with differences in Southern Europe where chickpea, soybean and vetch have smaller 10^th^ percentiles (in absolute values) compared to barley and sunflower (Supplementary Fig. 1). Confidence intervals obtained for estimated 10^th^ percentiles are larger than those calculated for standard deviations. Species rankings based on this risk measure are thus less reliable. Rankings derived from the third risk measure (expected yield loss, hereby defined as the mean value of yield anomalies below the 10^th^ percentile) are almost identical to those derived from yield standard deviations (Supplementary Fig. 2). The only notable difference is observed for Southern Europe bean ranks: 1^st^ according to yield standard deviation computed using the polynomial regression technique and 6^th^ for expected yield loss. Note, however, that bean is also ranked 6^th^ when yield standard deviation is calculated using the local regression. Expected yield losses are greater than 0.25 tons for several legume crops: lupin and soybean in Western Europe, lupin, bean, vetch and fababean in Northern Europe, chickpea, soybean, vetch and bean (both not with local regression) in Southern Europe and all legume species in Eastern Europe (Supplementary Fig. 2). In the above-mentioned cases, expected yield losses are equivalent to more than 25% of expected yield values estimated by fitted yield trends. The highest yield loss values are obtained for lupin; for this species, expected yield losses range from 0.37 to 0.57 tons depending on the region and detrending method used to compute yield anomalies. In comparison, expected yield loss values are much lower for wheat (i.e., 0.09 to 0.20 tons depending upon the region, Supplementary Fig. 2).

### Risk levels of legume and non-legume crops are smaller in the Americas

**Figure 3:**
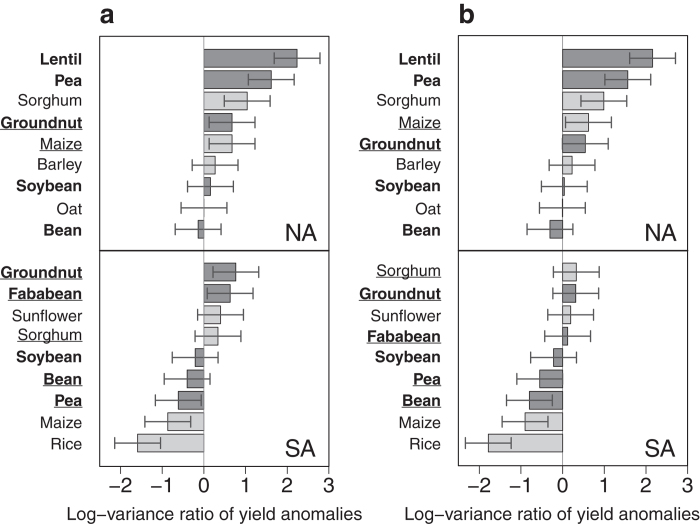
Log-variance ratio of yield anomalies for 9 crops compared to wheat in two American regions. Variances are calculated over 1961–2013 and log-transformed in North America (NA) and South America (SA). Yield anomalies are calculated using both regression models (polynomial (**a**), loess (**b**)). Horizontal bars correspond to 95% confidence intervals. Among the 9 crops, 5 are legume crops (bold names and dark grey bars) and 4 are non-legume crops (non-bold names and light grey bars). All crops are ranked according to variance ratio values (decreasing order). Crops that change in ranking between the polynomial and loess regression models used are underlined.

**Figure 4:**
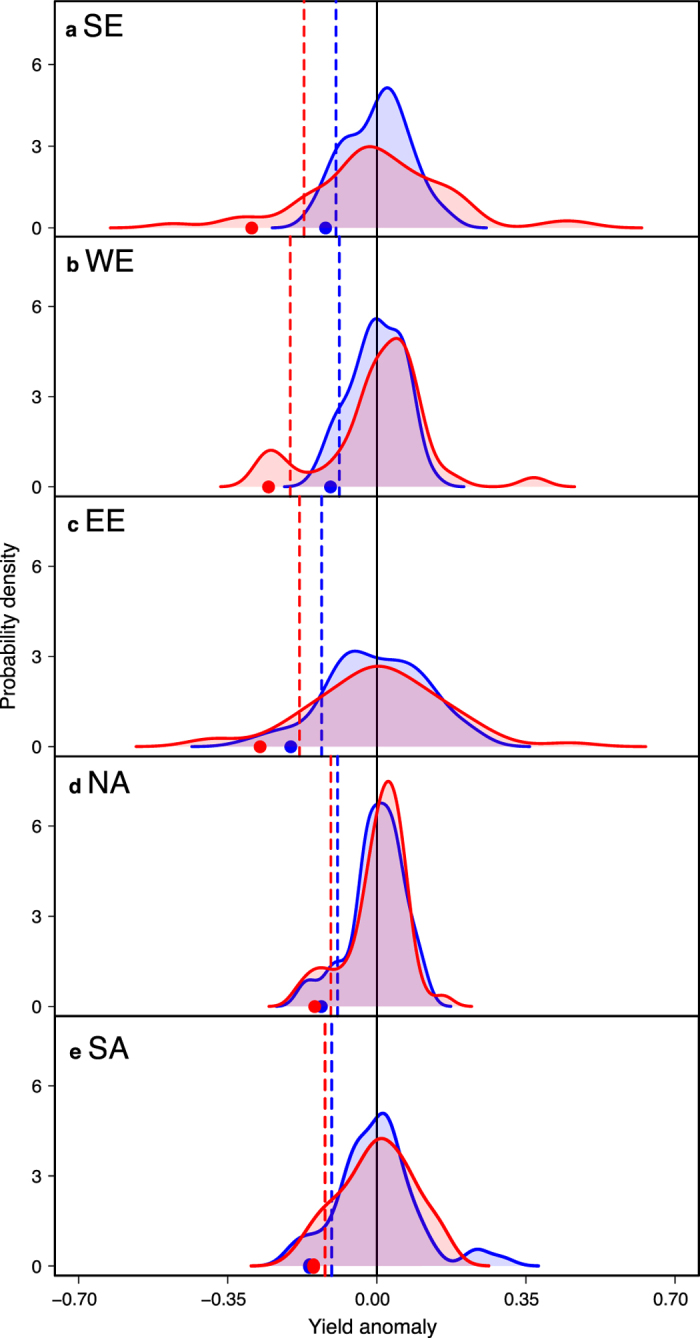
Yield anomaly distributions, 10^th^ percentile and expected yield loss for wheat and soybean in Europe and the Americas over 1961-2013. Probability densities of yield anomalies (curves), 10^th^ percentiles of yield anomalies (vertical dashed lines), and expected yield losses (mean values of yield anomalies lower than 10% percentiles; points) for wheat (in blue) and soybean (in red) in three European regions (i.e., Southern Europe (SE), Western Europe (WE) and Eastern Europe (EE)) and two American regions (i.e., North America (NA) and South America (SA)). Yield anomalies (relative differences between yield data and yield trend) are calculated using the polynomial regression models over 1961–2013. Northern Europe is excluded due to insufficient soybean yield data. Regions are ranked according to variance ratio values of soybean yield anomalies compared to wheat (i.e., Southern Europe shows the highest soybean variance ratio compared to wheat). Probability densities are estimated using a Gaussian smoothing kernel.

In North America, crop species characterized both by the highest and the lowest yield standard deviations are two legume crops, namely lentil and bean (Fig. 1e). In South America, groundnut and sorghum have the highest standard deviations (calculated from polynomial and local regression respectively). In the latter region, the species showing the lowest standard deviation is rice (Fig. 1f). In the Americas, the comparison between legume and non-legume species is in fact less disadvantageous for legumes. In South (North) America, only two (three) legume species show significantly higher yield variance compared to wheat (lentil, pea and groundnut in North America, and groundnut and fababean in South America, Fig. 3a,b). Importantly, the variance of soybean is close to or not significantly higher than wheat variance in both American regions (Figs. 1e,f and 3a,b). The variance of soybean is also lower than that of maize in North America, but higher in South America (Figs. 1e,f and 3a,b). Pea, maize, and rice show significantly lower yield variance than wheat in South America (Figs. 1f and 3a,b). Overall, whatever the risk measure considered or the detrending method, rankings of grain legumes are never significantly lower than those of non-legumes in either North or South America (*P* > 0.20) (Fig. 1e,f and Supplementary Figs. 1e, f and 2e, f). In fact, in the Americas, soybean risk measures are roughly equal to those calculated for wheat (Figs. 1e,f and 4d,e, Supplementary Figs. 1e,f and 2e,f). Hence, expected losses range from 0.13 to 0.15 tons for soybean, and from 0.13 to 0.16 tons for wheat, depending on the region and the detrending method. The small differences between the estimated risk levels of soybean and wheat are due to the strong similarities in yield anomaly distributions for these two species; in both North and South America, yield anomaly distributions obtained for soybean and wheat are almost the same (Fig. 4d,e). Consequently, soybean and wheat also have very similar values for 10^th^ percentile and expected yield losses. In Europe, soybean yield anomaly distributions have longer tails (revealing more extreme yield anomalies) than wheat distributions. This explains the contrasted 10^th^ percentile and expected yield loss values observed between soybean and wheat (Fig. 4a–c). Consistent results are obtained using the loess regression method (Supplementary Fig. 3).

### Higher volumes of risky crops tend to be grown on small proportions of total cultivated area

**Figure 5:**
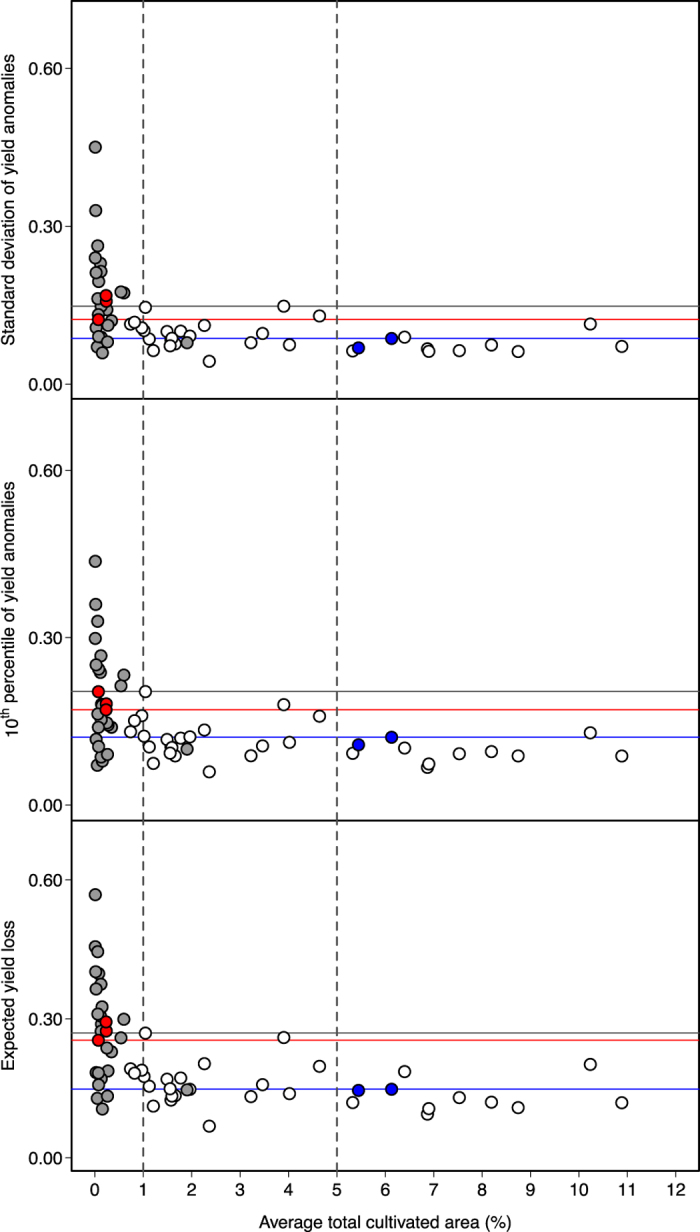
Yield risk measures as a function of the percentage of total cultivated areas in Europe and the Americas over 1961–2013. Risk measures are calculated over 1961–2013: standard deviation (**a**), 10^th^ percentile (absolute values, **b**) and expected yield loss (absolute values, **c**) for all crop*region combinations. Crop*region combinations corresponding to legume and non-legume crops are indicated by grey points and empty points, respectively. The percentages reported in the x-axis correspond to the percentage of total cultivated area by a given crop in a given region over 1961–2013. Soybeans grown in the Americas and Europe are indicated in blue and red points. Blue horizontal lines represent the maximal values of risk measures for soybean grown in the Americas. Red horizontal lines represent the minimal values of risk measures for soybean grown in Europe. Grey horizontal lines represent the maximal values of risk measures for non-legume crops. Grey vertical dashed lines represent the thresholds of 1% and 5% of total cultivated area. Yield anomalies are computed using the polynomial regression.

All species*region combinations with standard deviation above 0.15, with 10^th^ percentile above 0.20 or with expected yield loss above 0.27 are grain legumes (Fig. 5). Consistent results are obtained using the loess regression method (Supplementary Fig. 4). The proportions of total cultivated areas reported in Fig. 5 indicate that these species*region combinations are allocated to less than 1% of regional cultivated area. This is true for all three risk measures. Figure 5 also shows that species*region combinations allocated to more than 5% of the cultivated area are all characterized by low levels of risk (e.g., soybean in North and South America, Fig. 5). Our results show that species with higher risk levels (mostly legumes) are grown on small proportions of total cultivated area, whereas species allocated to high proportions of total cultivated area tend to be characterized by low levels of risk. Hence, there is a consistent pattern in the proportion of risk-cultivated area. However, several species (both legumes and non-legumes) characterized by low risk levels are also allocated to less than 1% of cultivated area. A low level of risk is clearly not a sufficient condition to insure a large proportion of cultivated area.

## Discussion

Based on European and American historical yield anomalies over the past five decades, we show that yields of legume crops are in general more variable than yields of non-legume crops in Europe, especially in Western, Eastern and Northern Europe. Our results also reveal that the levels of yield variability differ strongly across legume species. In the Americas, legume species also show the highest levels of yield variability, but the differences between legume and non-legume species are smaller. The trend-removal method (polynomial regression or local regression) used to calculate yield anomalies only marginally affects species rankings but has an effect on estimated values of yield standard deviations: yield anomalies and their standard deviations tend to be smaller when they are estimated using the local regression than when they are estimated with the polynomial regression (Fig. 1). The effect of trend-removal method on estimated yield standard deviations and other risk measures is small in most cases, with the exception of bean in Eastern and Southern Europe. We also investigated the sensitivity of our results to the length of yield time series. All three risk measures were computed over a more recent time period (1983-2013) and the results (Supplementary Figs. 5–8) are, in most cases, very similar to those obtained over the full time period (1961-2013). Reducing the time period increases to some extent lupin yield variance estimates in Western Europe and decreases those of soybean in Southern Europe.

This study is the first quantification of yield risk levels of a large range of legume species compared to non-legume species. Previous studies have used qualitative approaches based on farmers interviews and surveys^12^,^14^,^16^. Some studies have addressed variability of inter-annual market prices and incomes^12^,^15^,^22^,^23^. Although our assessment does not take important economic factors (e.g., crop prices, subsidies) into account, our results may help decision makers in defining targeted policies to support growing legumes in Europe. For example, yield insurance systems could be set up in order to compensate high yield losses for risky crops^24^,^25^,^26^. Such systems require accurate estimations of yield anomaly distributions in order to derive reliable risk premiums^17^,^18^,^19^,^20^. Our study indicates that, in Europe, risk premiums are likely to be higher for most of the legume species compared to major non-legume species. Note though that the use of aggregated yield data at the scale of large regions may under-estimate yield risk measures derived from less spatially aggregated levels (e.g., from farm data to country data). The larger the scale of yield aggregation, the more likely the effects of factors affecting crop yield may be averaged out, resulting in lower variability and risk of yield loss^27^,^28^.

The high susceptibility of biological nitrogen fixation – a function specific to legume crops – to both biotic^29^ and abiotic stresses^30^,^31^ could be a possible explanation to the observed higher variability of grain legume compared to non-legume yields. However, legume crops do not show the same levels of risk in Europe and in the Americas. Our results show that growing soybean entails lower risks in the Americas than in Europe (Figs. 2, 3 and 4). A broad spectrum of factors may be invoked to explain this difference from the use of specifically adapted varieties in the Americas to the effects of climate variability or pests and diseases. Evidently, there are major differences in cropping systems between the studied regions. The wider use of genetically modified soybean varieties in North and South America is an important feature of these differences and may contribute to the observed risk contrasts, as reported for some non-legume crops^32^. Recent studies outline a comparative lack of breeding investment in Europe to improve grain legume adaptation to local agroclimatic conditions and management techniques^11^,^16^,^33^,^34^, such as crop protection or sowing and harvesting techniques^35^,^36^,^37^. Note that a general lack of specific agronomic references to manage grain legumes may be a barrier for farmers cultivating these crops in Europe^11^,^16^. Increasing our knowledge about how grain legumes respond to soil and the environment will improve management of these crops across a large diversity of European agroclimatic zones.

We failed to find any clear relationship between the average yield annual growth rates - sometimes proposed as a proxy for technological improvments - and yield variance for legume and non-legume crops (Supplementary Figs. 9 and 10). At the scale of large world regions, it is thus not possible to conclude on a relationship between risk and yield trends. Interestingly, our results indicate that legume species showing high levels of yield variability are always allocated to less than 1% of cultivated areas, suggesting that high yield variability may restrict the expansion of grain legume crops in Europe. Nonetheless, many low-risk crops are cultivated on less than 1% of agricultural areas. Variability in yields is thus only one of the factors behind the variations in cultivated areas. Other external factors may explain this limited expansion, for example, the historical dependence to the Common Agricultural Policy measures^12^; the low level of production of processed products^11^,^38^; and the competition with soybean imports from the Americas^15^ or protein-rich by-products derived from non-legume crops^38^,^39^. It may also be that European farmers are not incentivized to cultivate these species on soils of good quality in adequate environments, and that they prefer growing more profitable major crops in these environments (e.g., wheat, maize and rapeseed)^11^,^16^,^21^.

In average over the 1961–2011 period, Europe imported 63% of its grain legume domestic supply^2^. The European livestock sector dependency on American soybean imports is leading the EU to investigate alternative paths to sustain its meat production^1^,^38^,^39^. This could partly be achieved by cropping significantly more legumes in Europe^11^,^15^, i.e., reducing risk aversion of European farmers for growing legume species. In the coming years, investments in legume breeding and in legume-based cropping system design will be unavoidable if we want to improve the plant protein supply and demand balance in Europe.

## Methods

### Yield data

Yield time series were retrieved from the Food and Agriculture Organization statistics (FAOSTAT) database^2^. These data had already been used in several studies for analyzing past yield trends and anomalies^40^,^41^,^42^,^43^. Crop yield time series were collected from four European regions (i.e., Western, Eastern, Northern and Southern Europe) and for two American regions (i.e., North and South America). Supplementary Table 1 lists countries belonging to the studied regions and aggregated by the United Nations Statistics Division. In general, the database time interval spans from 1961 to 2013 (i.e., *n* = 53 years). There are only two exceptions, for which time intervals are shorter (i.e., soybean in Western Europe from 1973 to 2013 with *n* = 41 years and lupin in Northern Europe from 1992 to 2013 with *n* = 22 years). For each region, we selected the five most cropped legume and non-legume species in terms of average harvested area over 1961-2013. A FAOSTAT crop item is defined as a legume when the crop item species belongs to the family of Fabaceae plants, according to the United States Department of Agriculture classification^44^. Concerning grain legumes, we excluded FAOSTAT crop items for which (i) different legume species are pooled into one single crop item (i.e., ‘Pulses, nes’ and ‘String beans’ crop items) and (ii) the post-harvest use is for shelling (i.e., ‘Beans, green’, ‘Peas, green’ and ‘Vegetables, leguminous nes’ crop items). Regarding non-legumes, we excluded FAOSTAT crop items that correspond to vegetables and fruits (e.g., ‘Grape’ and ‘Olive’ crop items in Southern Europe). For each region, Supplementary Table 2 documents (i) the 10 crop species examined, (ii) the number of observed FAOSTAT yields over 1961-2013 and (iii) the average harvested area in decreasing order.

For each crop and region, a yield trend is estimated using polynomial and local ‘loess’ regression models fitted to observed data (thereafter, polynomial and loess regression models respectively). The polynomial regression model groups three variants which are linear, quadratic and cubic as follows:


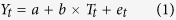



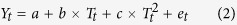



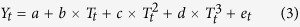


where *Y*_*t*_ is the yield data in year *t* (t ha^-1^) and *t* is the year index (with *t* = 1 for the first year of the time series, i.e., 1961). The letters *a*, *b*, *c* and *d* are parameters of the polynomial yield trend, and *e*_*t*_ is the residual error which equals the difference between *Y*_*t*_ and the polynomial trend. The three polynomial variants are systematically fitted to time series using the ‘lm’ in the R software version 3.0.2^45^ and compared with the Akaike Information Criteria (AIC)^46^. The polynomial variant that has the lowest AIC is selected. In addition, we used the loess regression model to assess the suitability of a different calculation method for analyzing yield time series. We used the ‘loess’ function in the R software with standard arguments (span = 0.75, degree = 2) (Supplementary Fig. 11). The selected polynomial model and the output of the local ‘loess’ regression are both used to calculate a yield trend further noted 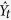. In a second step, a yield anomaly 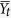 is defined as a normalized yield residual, which is obtained by the difference between yield data *Y*_*t*_ and expected yields 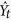 (i.e., yield residual) and then normalized with 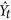:


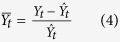


We thus obtained time series of yield anomalies describing yield variability. Normalizing yield residual enables us to compare crop species and regions with no dimension dependency.

### Estimation of yield risk measures

To survey yield variability leading to yield loss of legume crops, a set of three classic risk measures^18^,^19^,^24^,^47^,^48^ is evaluated across time series distribution of yield anomalies. The first risk measure is standard deviation expressed as:


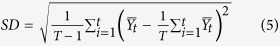


Yield variance is the squared value of the standard deviation. The second risk measure is the 10^th^ percentile defined by:


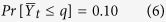


where *q* is the 10^th^ percentile of the yield anomalies distribution. It accounts for the value beyond which the yield is considered as a substantial loss. Values of *q* are estimated from the yield anomalies using the ‘quantile’ function in the R software. It would have been difficult to estimate more extreme quantiles (e.g., 5^th^ percentile) due to the relatively short yield length of the time series. Supplementary Figure 1 shows that confidence intervals of the estimated 10^th^ percentiles are large and the situation would have been even worst with the 5^th^ quantile. The third risk measure is the expected yield loss^19^,^47^. It is equal to the mean of yield anomalies that are lower than the 10^th^ percentile *q* and is calculated as follows:


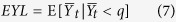


It represents the yield loss that is expected to exceed *q* with 1 chance of 10. For each of the three risk measures (standard deviation of yield anomalies, 10^th^ percentile of yield anomalies, expected yield loss), we calculated the 95% confidence intervals by bootstrap iterations with 10,000 samples. Crop species are then ranked from the highest to the lowest value, using each risk measure in turn. Within a single classification, we then tested the hypothesis that rankings of legume crops are significantly higher than rankings of non-legume crops. This test is performed using the Wilcoxon rank test with the ‘wilcox.test’ function in the R software.

For each species and each region, we calculated the variance ratio of yield anomalies of a given crop to wheat. We selected wheat as a reference crop in the variance ratio as it is the non-legume crop with the lowest risk measures in three out of four European regions and in North America over the 1961–2013 period. To test whether variance of yield anomalies of a given crop is different from wheat, we used the ‘variance.test’ function in the R software (α = 0.05). The 95% confidence intervals are obtained from the calculations of this function. To appraise the robustness of our calculations of risk measures and of crop rankings, we provided results for a shorter time period that begins in 1983 and ends in 2013 (i.e., *n* = 31 years). Note that, however, expected yield loss measure was not provided for this shorter time period due to an insufficient number of yield anomalies below the 10^th^ percentile in order to calculate accurately their mean. We calculated the average cultural area of each crop in each region, expressed in percentage of total cultivated area calculated from all FAO items averaged over 1961–2013. We finally calculated the average annual growth rate of yield for each crop in each region over 1961–2013 (i.e., *n* = 53 years), expressed in percentage and using the fitted yield trend 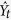 computed from both polynomial and loess regression methods:


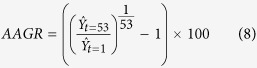


where *AAGR* is the average annual growth rate of yield expressed in percentage, 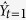 and 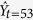 are the yield trends in 1961 and 2013, respectively.

## Additional Information

**How to cite this article**: Cernay, C. *et al.* Estimating variability in grain legume yields across Europe and the Americas. *Sci. Rep.* **5**, 11171; doi: 10.1038/srep11171 (2015).

## Supplementary Material

Supplementary Information

Supplementary Data 1

Supplementary Data 2
