# Supplementary material for: Estimating variability in grain legume yields across Europe and the Americas
Source: Sci Rep. 2015 Jun 8;5:11171. doi: 10.1038/srep11171 (PMC4459183; doi:10.1038/srep11171)
Supplement: Supplementary Information [file srep11171-s1.pdf]

Supplementary Information

Estimating variability in grain legume yields across Europe and the Americas

Charles Cernay[1*], Tamara Ben-Ari[1], Elise Pelzer[1], Jean-Marc Meynard[3,4] & David Makowski[1,2]

[1]INRA, UMR 211 Agronomie, F–78850, Thiverval–Grignon, France

[2]AgroParisTech, UMR 211 Agronomie, F–78850, Thiverval–Grignon, France

[3]INRA, UMR 1018 Sciences pour l'Action et le Développement: Activités, Produits, Territoires, F–78850, Thiverval–Grignon, France

[4]AgroParisTech, UMR 1018 Sciences pour l'Action et le Développement: Activités, Produits, Territoires, F–78850, Thiverval–Grignon, France

[*]Correspondence: charles.cernay@grignon.inra.fr

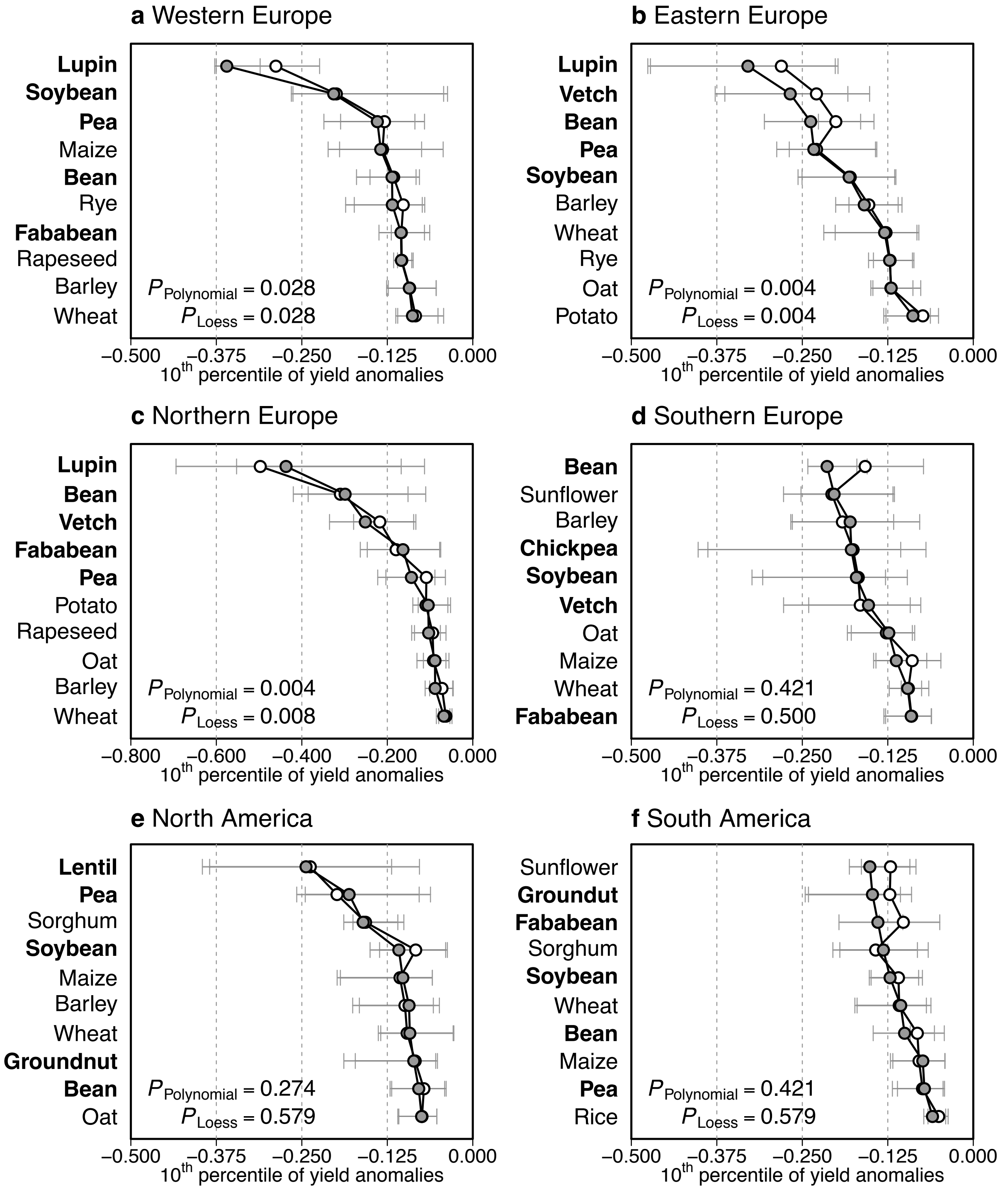
a Western Europe
Lupin
Soybean
Pea
Maize
Bean
Rye
Fababean
Rapeseed
Barley
Wheat
PPolynomial = 0.028
PLoess = 0.028
−0.500 −0.375 −0.250 −0.125 0.000
10th percentile of yield anomalies
b Eastern Europe
Lupin
Vetch
Bean
Pea
Soybean
Barley
Wheat
Rye
Oat
Potato
PPolynomial = 0.004
PLoess = 0.004
−0.500 −0.375 −0.250 −0.125 0.000
10th percentile of yield anomalies
c Northern Europe
Lupin
Bean
Vetch
Fababean
Pea
Potato
Rapeseed
Oat
Barley
Wheat
PPolynomial = 0.004
PLoess = 0.008
−0.800 −0.600 −0.400 −0.200 0.000
10th percentile of yield anomalies
d Southern Europe
Bean
Sunflower
Barley
Chickpea
Soybean
Vetch
Oat
Maize
Wheat
Fababean
PPolynomial = 0.421
PLoess = 0.500
−0.500 −0.375 −0.250 −0.125 0.000
10th percentile of yield anomalies
e North America
Lentil
Pea
Sorghum
Soybean
Maize
Barley
Wheat
Groundnut
Bean
Oat
PPolynomial = 0.274
PLoess = 0.579
−0.500 −0.375 −0.250 −0.125 0.000
10th percentile of yield anomalies
f South America
Sunflower
Groundut
Fababean
Sorghum
Soybean
Wheat
Bean
Maize
Pea
Rice
PPolynomial = 0.421
PLoess = 0.579
−0.500 −0.375 −0.250 −0.125 0.000
10th percentile of yield anomalies

Supplementary Figure 1: 10th percentile of yield anomalies for 10 crops in Europe and the Americas over 1961-2013.

Values of 10th percentile of yield anomalies for 10 crops in four European and two American regions over 1961-2013. Polynomial (grey points) and local 'loess' (empty points) regression models are used to calculate yield anomalies (relative differences between yield data and yield trend). Horizontal lines correspond to 95% confidence intervals estimated by bootstrap (10,000 samples). Among the 10 crops, 5 are legume crops (bold names) and 5 are non-legume crops (non-bold). All crops are ranked according to standard deviation of yield anomalies calculated using the polynomial model (decreasing order). $P_{\text{Polynomial}}$ and P_{Loess} correspond to the p-value of the Wilcoxon rank test on a hypothesis assuming that ranks of legume and non-legume crops do not differ (against the alternative that legume crop ranking is lower) and are computed using the polynomial and loess regression respectively. The number of yield data includes 53 observations for most of crops and regions. There are two exceptions: soybean yield data in Western Europe includes 41 observations and lupin yield data in Northern Europe includes 22 observations. Grey vertical dashed lines represent the 10th percentile values of -0.375, -0.250, -0.125 in all regions but -0.600, -0.400 and -0.200 in Northern Europe.

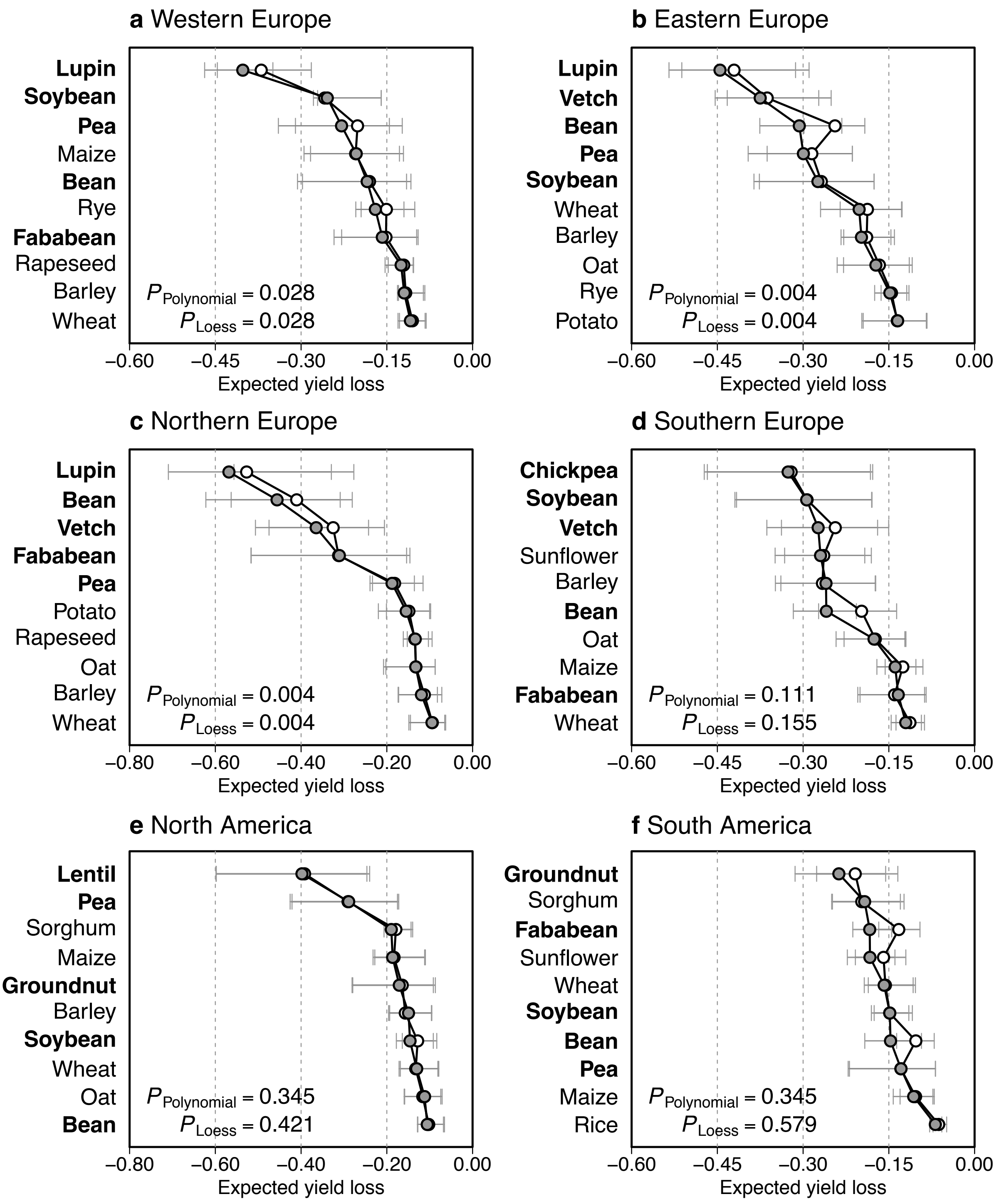
a Western Europe
Lupin
Soybean
Pea
Maize
Bean
Rye
Fababean
Rapeseed
Barley
Wheat
$P_{Polynomial}$ = 0.028
P_{Loess} = 0.028
−0.60 −0.45 −0.30 −0.15 0.00
Expected yield loss
b Eastern Europe
Lupin
Vetch
Bean
Pea
Soybean
Wheat
Barley
Oat
Rye
Potato
$P_{Polynomial}$ = 0.004
P_{Loess} = 0.004
−0.60 −0.45 −0.30 −0.15 0.00
Expected yield loss
c Northern Europe
Lupin
Bean
Vetch
Fababean
Pea
Potato
Rapeseed
Oat
Barley
Wheat
$P_{Polynomial}$ = 0.004
P_{Loess} = 0.004
−0.80 −0.60 −0.40 −0.20 0.00
Expected yield loss
d Southern Europe
Chickpea
Soybean
Vetch
Sunflower
Barley
Bean
Oat
Maize
Fababean
Wheat
$P_{Polynomial}$ = 0.111
P_{Loess} = 0.155
−0.60 −0.45 −0.30 −0.15 0.00
Expected yield loss
e North America
Lentil
Pea
Sorghum
Maize
Groundnut
Barley
Soybean
Wheat
Oat
Bean
$P_{Polynomial}$ = 0.345
P_{Loess} = 0.421
−0.80 −0.60 −0.40 −0.20 0.00
Expected yield loss
f South America
Groundnut
Sorghum
Fababean
Sunflower
Wheat
Soybean
Bean
Pea
Maize
Rice
$P_{Polynomial}$ = 0.345
P_{Loess} = 0.579
−0.60 −0.45 −0.30 −0.15 0.00
Expected yield loss

Supplementary Figure 2: Expected yield loss for 10 crops in Europe and the Americas over 1961-2013.

Expected yield loss (mean values of yield anomalies lower than the 10th percentile) for 10 crops in four European and two American regions over 1961-2013. Polynomial (grey points) and local 'loess' (empty points) regression models are used to calculate yield anomalies (relative differences between yield data and yield trend). Horizontal lines correspond to 95% confidence intervals estimated by bootstrap (10,000 samples). Among the 10 crops, 5 are legume crops (bold names) and 5 are non-legume crops (non-bold). All crops are ranked according to standard deviation of yield anomalies calculated using the polynomial model (decreasing order). $P_{Polynomial}$ and P_{Loess} correspond to the p-value of the Wilcoxon rank test on a hypothesis assuming that ranks of legume and non-legume crops do not differ (against the alternative that legume crop ranking is lower) and are computed using the polynomial and loess regression respectively. The number of yield data includes 53 observations for most of crops and regions. There are two exceptions: soybean yield data in Western Europe includes 41 observations and lupin yield data in Northern Europe includes 22 observations. Grey vertical dashed lines represent the values of expected yield loss of -0.45, -0.30, -0.15 in all regions but -0.60, -0.40 and -0.20 in Northern Europe and North America.

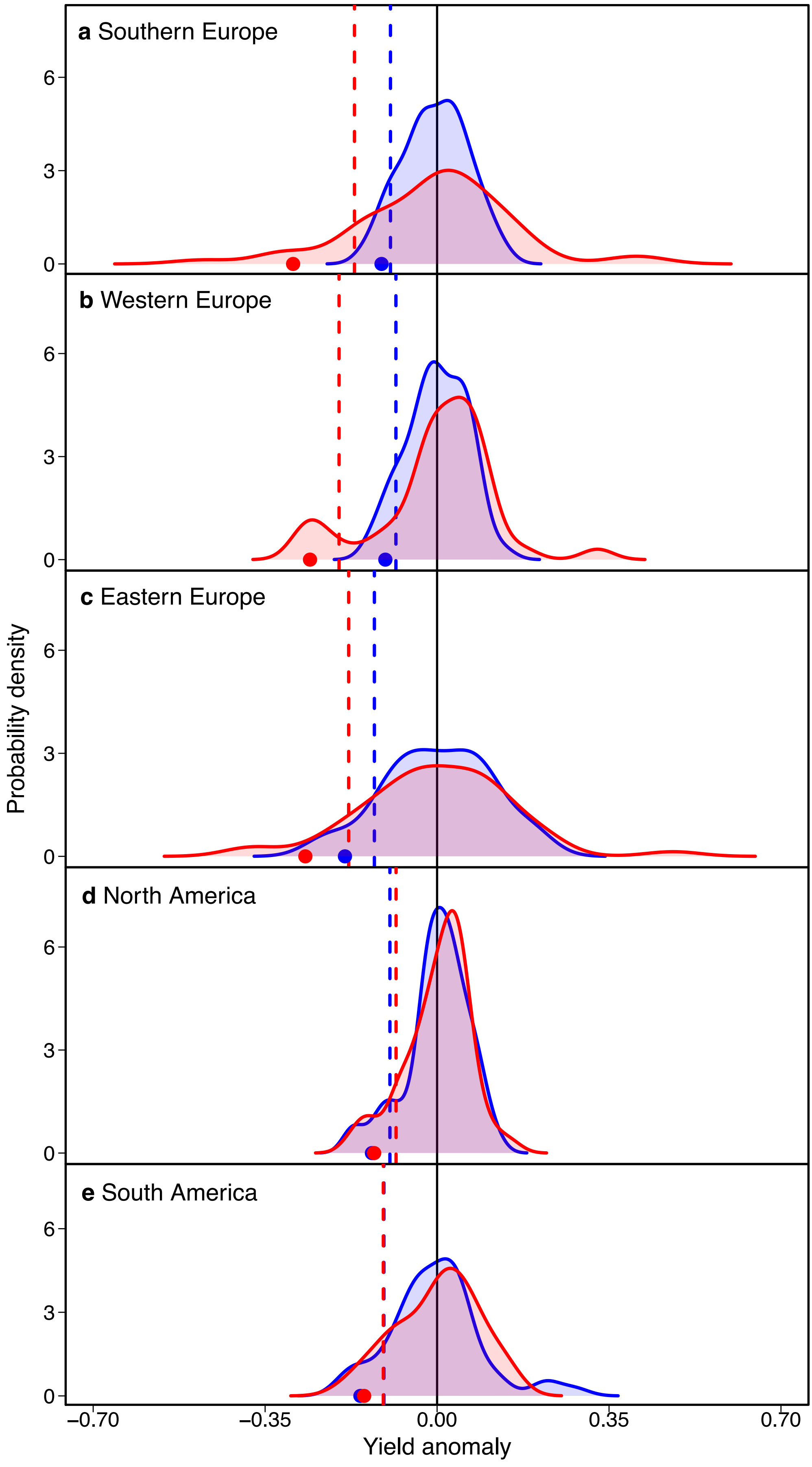
a Southern Europe
b Western Europe
c Eastern Europe
d North America
e South America
Probability density
0
3
6
−0.70
−0.35
0.00
0.35
0.70
Yield anomaly

Supplementary Figure 3: Yield anomaly distributions, 10th percentile and expected yield loss for wheat and soybean in Europe and the Americas over 1961-2013 using the loess regression.

Probability densities of yield anomalies (curves), 10th percentiles of yield anomalies (vertical dashed lines), and expected yield losses (mean values of yield anomalies lower than 10% percentiles; points) for wheat (in blue) and soybean (in red) in three European regions and two American regions. Yield anomalies (relative differences between yield data and yield trend) are calculated using the loess regression model over 1961-2013. Northern Europe is excluded due to insufficient soybean yield data. Regions are ranked according to the variance ratio of soybean yield anomalies compared to wheat (i.e., Southern Europe shows the highest soybean variance ratio compared to wheat). Probability densities are estimated using a Gaussian smoothing kernel.

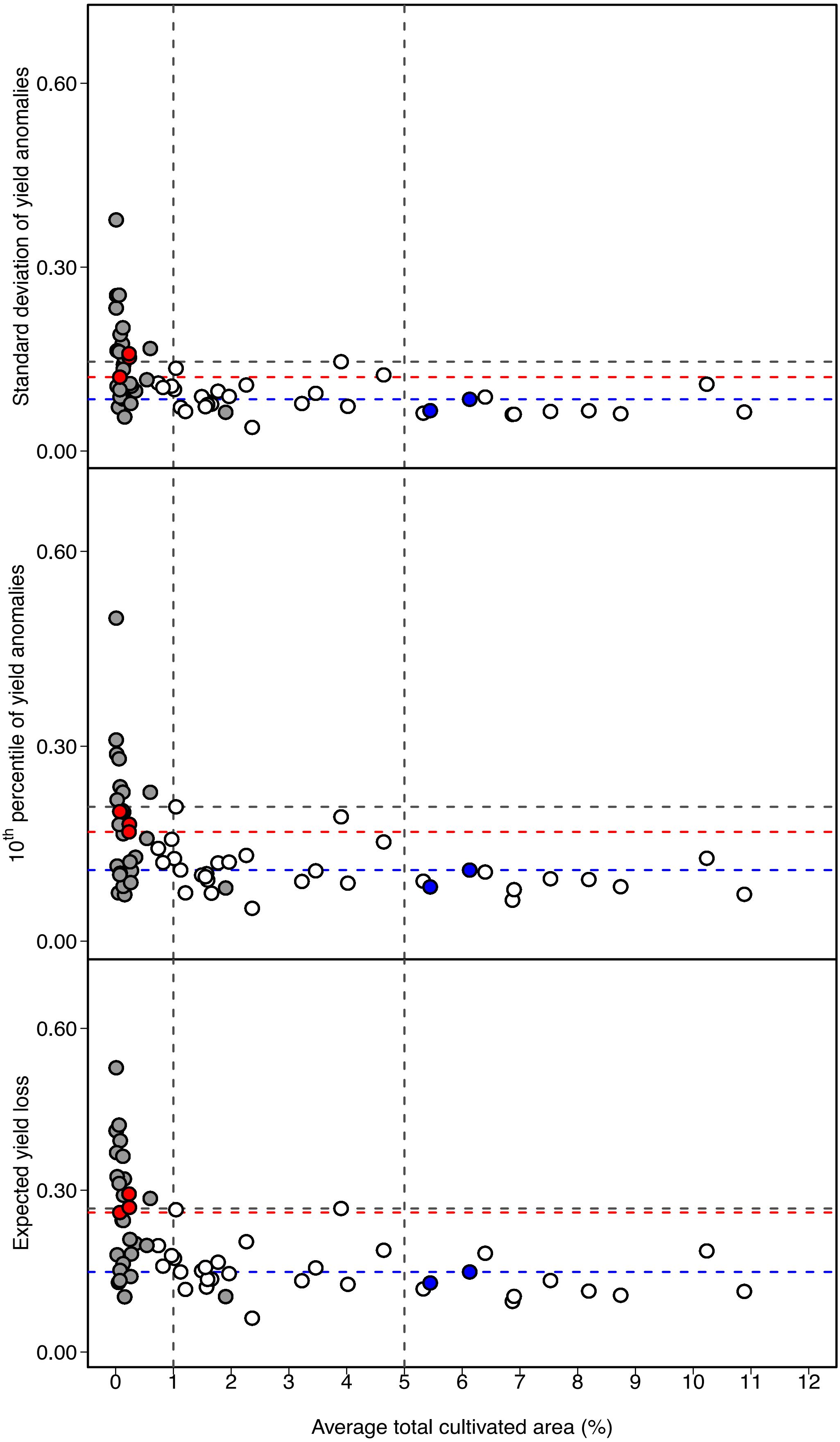

Standard deviation of yield anomalies
0.60
0.30
0.00
10th percentile of yield anomalies
0.60
0.30
0.00
Expected yield loss
0.60
0.30
0.00
0 1 2 3 4 5 6 7 8 9 10 11 12
Average total cultivated area (%)

Supplementary Figure 4: Yield risk measures as a function of the percentage of total cultivated areas in Europe and the Americas over 1961-2013 using the loess regression.

Risk measures are calculated over 1961-2013: standard deviation (a), 10th percentile (absolute values, b) and expected yield loss (absolute values, c) for all crop*region combinations. Crop*region combinations corresponding to legume and non-legume crops are indicated by grey points and empty points, respectively. The percentages reported in the x-axis correspond to the percentage of total cultivated areas by a given crop in a given region over 1961-2013. Soybeans grown in the Americas and Europe are indicated in blue and red points. Blue dashed horizontal lines represent the maximal values of risk measures for soybean grown in the Americas. Red dashed horizontal lines represent the minimal values of risk measures for soybean grown in Europe. Grey horizontal dashed lines represent the maximal values of risk measures for non-legumes. Grey vertical dashed lines represent the thresholds of 1% and 5% of total cultivated areas. Yield anomalies are computed using the loess regression.

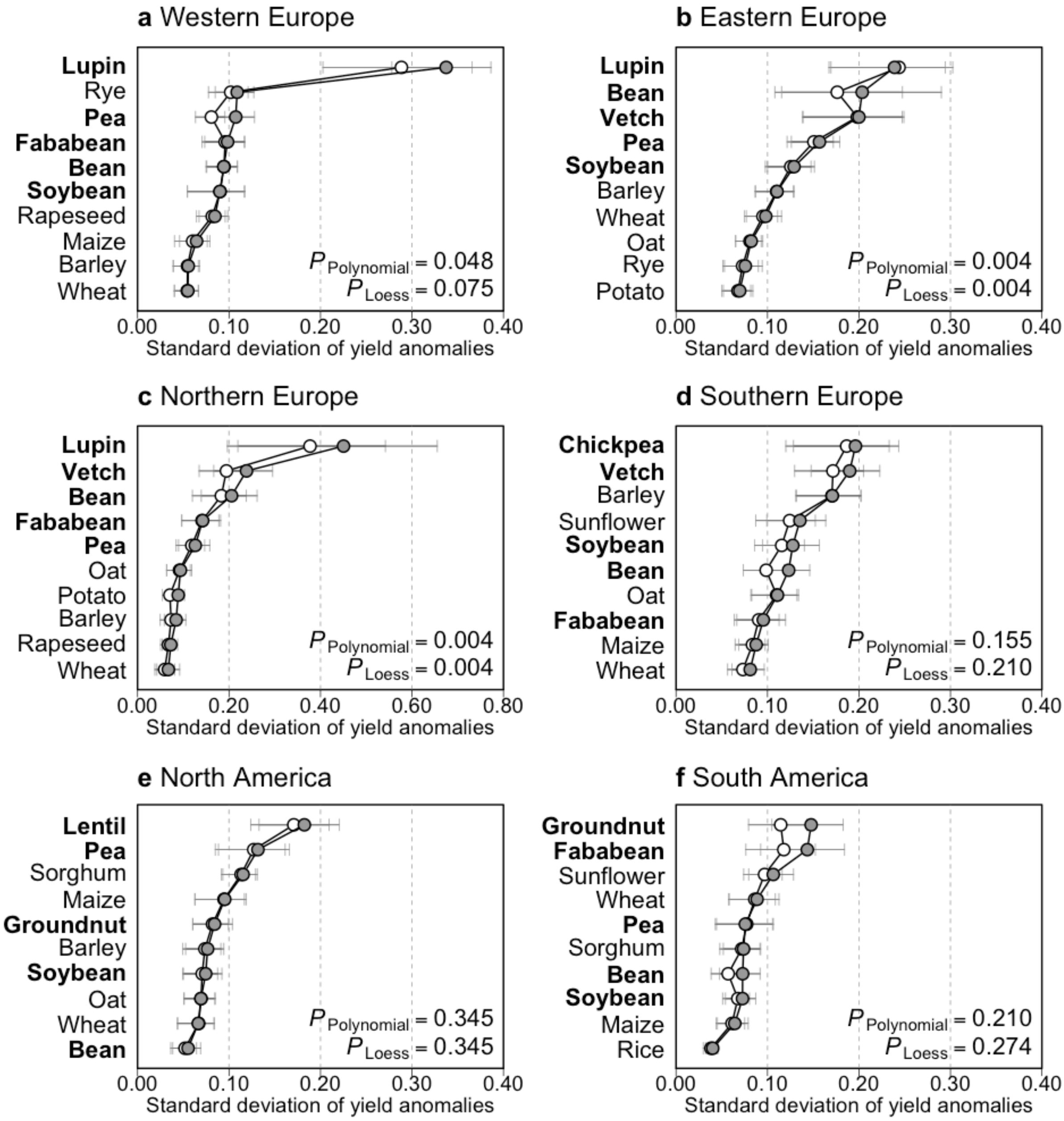
a Western Europe
Lupin
Rye
Pea
Fababean
Bean
Soybean
Rapeseed
Maize
Barley
Wheat
$P_{\text{Polynomial}} = 0.048$
$P_{\text{Loess}} = 0.075$
0.00 0.10 0.20 0.30 0.40
Standard deviation of yield anomalies
b Eastern Europe
Lupin
Bean
Vetch
Pea
Soybean
Barley
Wheat
Oat
Rye
Potato
$P_{\text{Polynomial}} = 0.004$
$P_{\text{Loess}} = 0.004$
0.00 0.10 0.20 0.30 0.40
Standard deviation of yield anomalies
c Northern Europe
Lupin
Vetch
Bean
Fababean
Pea
Oat
Potato
Barley
Rapeseed
Wheat
$P_{\text{Polynomial}} = 0.004$
$P_{\text{Loess}} = 0.004$
0.00 0.20 0.40 0.60 0.80
Standard deviation of yield anomalies
d Southern Europe
Chickpea
Vetch
Barley
Sunflower
Soybean
Bean
Oat
Fababean
Maize
Wheat
$P_{\text{Polynomial}} = 0.155$
$P_{\text{Loess}} = 0.210$
0.00 0.10 0.20 0.30 0.40
Standard deviation of yield anomalies
e North America
Lentil
Pea
Sorghum
Maize
Groundnut
Barley
Soybean
Oat
Wheat
Bean
$P_{\text{Polynomial}} = 0.345$
$P_{\text{Loess}} = 0.345$
0.00 0.10 0.20 0.30 0.40
Standard deviation of yield anomalies
f South America
Groundnut
Fababean
Sunflower
Wheat
Pea
Sorghum
Bean
Soybean
Maize
Rice
$P_{\text{Polynomial}} = 0.210$
$P_{\text{Loess}} = 0.274$
0.00 0.10 0.20 0.30 0.40
Standard deviation of yield anomalies

Supplementary Figure 5: Standard deviation of yield anomalies for 10 crops in Europe and the Americas over 1983-2013.

Standard deviation of yield anomalies for 10 crops in four European and two American regions over 1983-2013. Polynomial (grey points) and local 'loess' (empty points) regression models are used to calculate yield anomalies (relative differences between yield data and yield trend). Horizontal lines correspond to 95% confidence intervals estimated by bootstrap (10,000 samples). Among the 10 crops, 5 are legume crops (bold names) and 5 are non-legume crops (non-bold). All crops are ranked according to standard deviation of yield anomalies calculated using the polynomial model (decreasing order). $P_{\mathrm{Polynomial}}$ and P_{Loess} correspond to the p-value of the Wilcoxon rank test on a hypothesis assuming that ranks of legume and non-legume crops do not differ (against the alternative that legume crop ranking is lower) and are computed the polynomial and loess regression respectively. The number of yield data includes 31 observations for all crops and regions but lupin yield data in Northern Europe includes 22 observations. Grey vertical dashed lines represent the standard deviation values of 0.10, 0.20 and 0.30 in all regions but 0.20, 0.40 and 0.60 in Northern Europe.

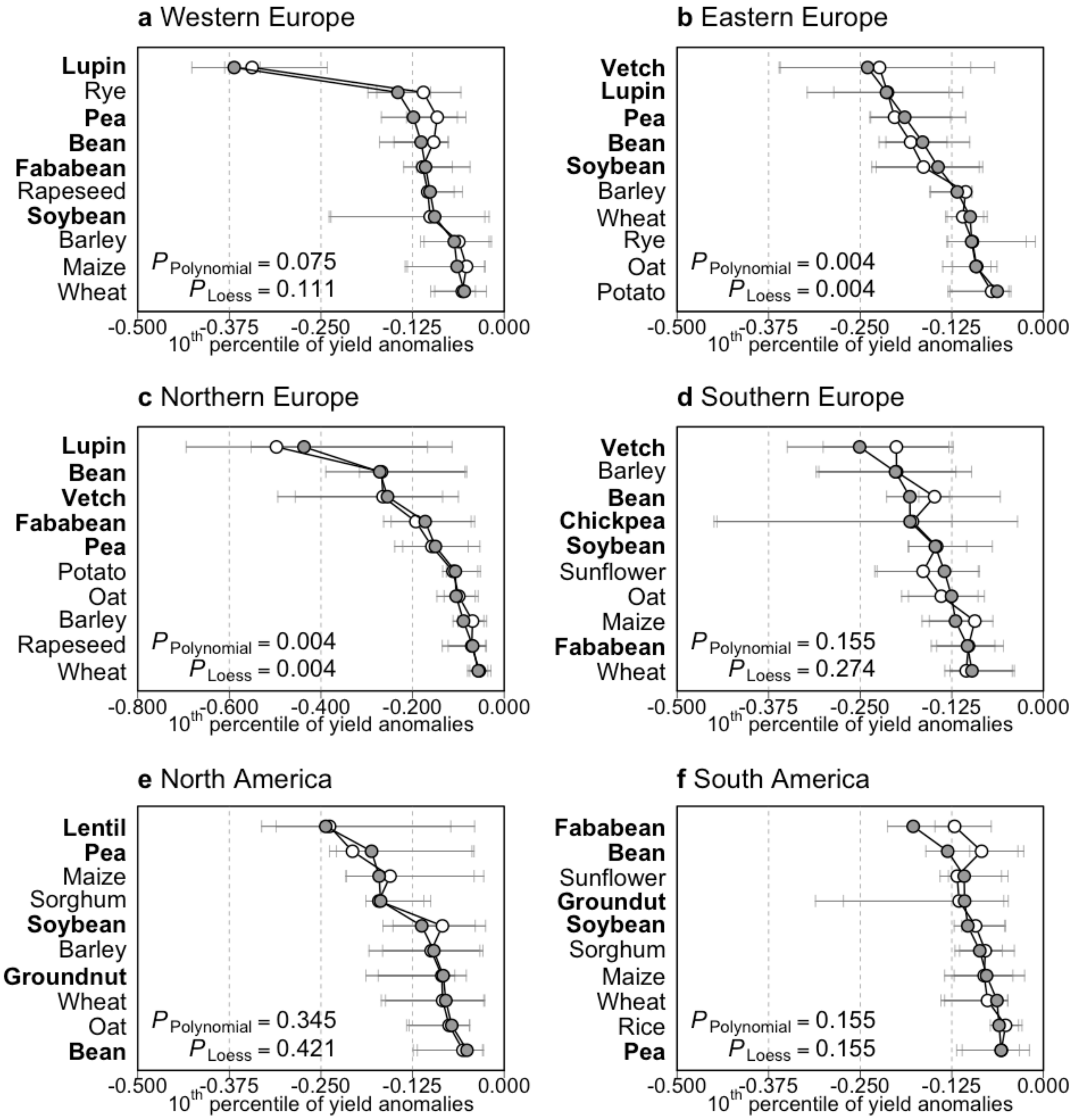
a Western Europe
Lupin
Rye
Pea
Bean
Fababean
Rapeseed
Soybean
Barley
Maize
Wheat
P Polynomial = 0.075
P Loess = 0.111
-0.500
-0.375
-0.250
-0.125
0.000
10th percentile of yield anomalies
b Eastern Europe
Vetch
Lupin
Pea
Bean
Soybean
Barley
Wheat
Rye
Oat
Potato
P Polynomial = 0.004
P Loess = 0.004
c Northern Europe
Lupin
Bean
Vetch
Fababean
Pea
Potato
Oat
Barley
Rapeseed
Wheat
P Polynomial = 0.004
P Loess = 0.004
-0.800
-0.600
-0.400
-0.200
0.000
d Southern Europe
Vetch
Barley
Bean
Chickpea
Soybean
Sunflower
Oat
Maize
Fababean
Wheat
P Polynomial = 0.155
P Loess = 0.274
e North America
Lentil
Pea
Maize
Sorghum
Soybean
Barley
Groundnut
Wheat
Oat
Bean
P Polynomial = 0.345
P Loess = 0.421
f South America
Fababean
Bean
Sunflower
Groundnut
Soybean
Sorghum
Maize
Wheat
Rice
Pea
P Polynomial = 0.155
P Loess = 0.155

Supplementary Figure 6: 10th percentile of yield anomalies for 10 crops in Europe and the Americas over 1983-2013.

Values of 10th percentile of yield anomalies for 10 crops in four European and two American regions over 1983-2013. Polynomial (grey points) and local 'loess' (empty points) regression models are used to calculate yield anomalies (relative differences between yield data and yield trend). Horizontal lines correspond to 95% confidence intervals estimated by bootstrap (10,000 samples). Among the 10 crops, 5 are legume crops (bold names) and 5 are non-legume crops (non-bold). All crops are ranked according to standard deviation of yield anomalies calculated using the polynomial model (decreasing order). $P_{\text{Polynomial}}$ and P_{Loess} correspond to the p-value of the Wilcoxon rank test on a hypothesis assuming that ranks of legume and non-legume crops do not differ (against the alternative that legume crop ranking is lower) and are computed using the polynomial and loess regression respectively. The number of yield data includes 31 observations for all crops and regions but lupin yield data in Northern Europe includes 22 observations. Grey vertical dashed lines represent the standard deviation values of 0.10, 0.20 and 0.30 in all regions but 0.20, 0.40 and 0.60 in Northern Europe.

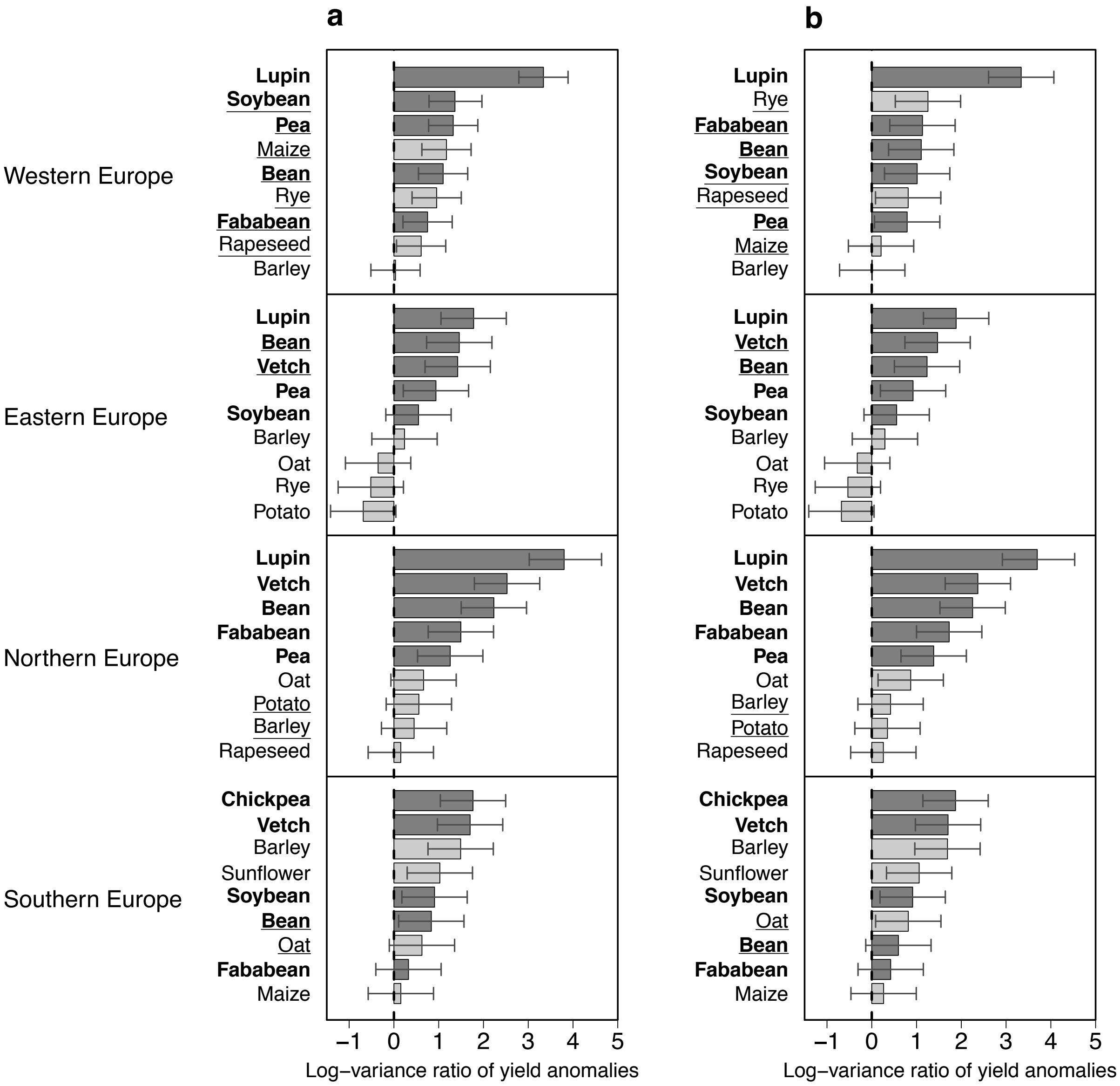
a
b
Western Europe
Eastern Europe
Northern Europe
Southern Europe
Lupin
Soybean
Pea
Maize
Bean
Rye
Fababean
Rapeseed
Barley
Lupin
Bean
Vetch
Pea
Soybean
Barley
Oat
Rye
Potato
Lupin
Vetch
Bean
Fababean
Pea
Oat
Potato
Barley
Rapeseed
Chickpea
Vetch
Barley
Sunflower
Soybean
Bean
Oat
Fababean
Maize
Lupin
Rye
Fababean
Bean
Soybean
Rapeseed
Pea
Maize
Barley
Lupin
Vetch
Bean
Pea
Soybean
Barley
Oat
Rye
Potato
Lupin
Vetch
Bean
Fababean
Pea
Oat
Barley
Potato
Rapeseed
Chickpea
Vetch
Barley
Sunflower
Soybean
Oat
Bean
Fababean
Maize
−1 0 1 2 3 4 5
Log−variance ratio of yield anomalies
−1 0 1 2 3 4 5
Log−variance ratio of yield anomalies

Supplementary Figure 7: Log-variance ratio of yield anomalies for 9 crops compared to wheat in four European regions over 1983-2013.

Variances are calculated over 1983-2013 and log-transformed. Yield anomalies are calculated using both regression models (polynomial (a), loess (b)). Horizontal lines correspond to 95% confidence intervals. Among the 9 crops, 5 are legume crops (bold names and dark grey bars) and 4 are non-legume crops (non-bold names and light grey bars). All crops are ranked according to variance ratio values (decreasing order). Crops that change of ranking between polynomial and loess regression models used are underlined.

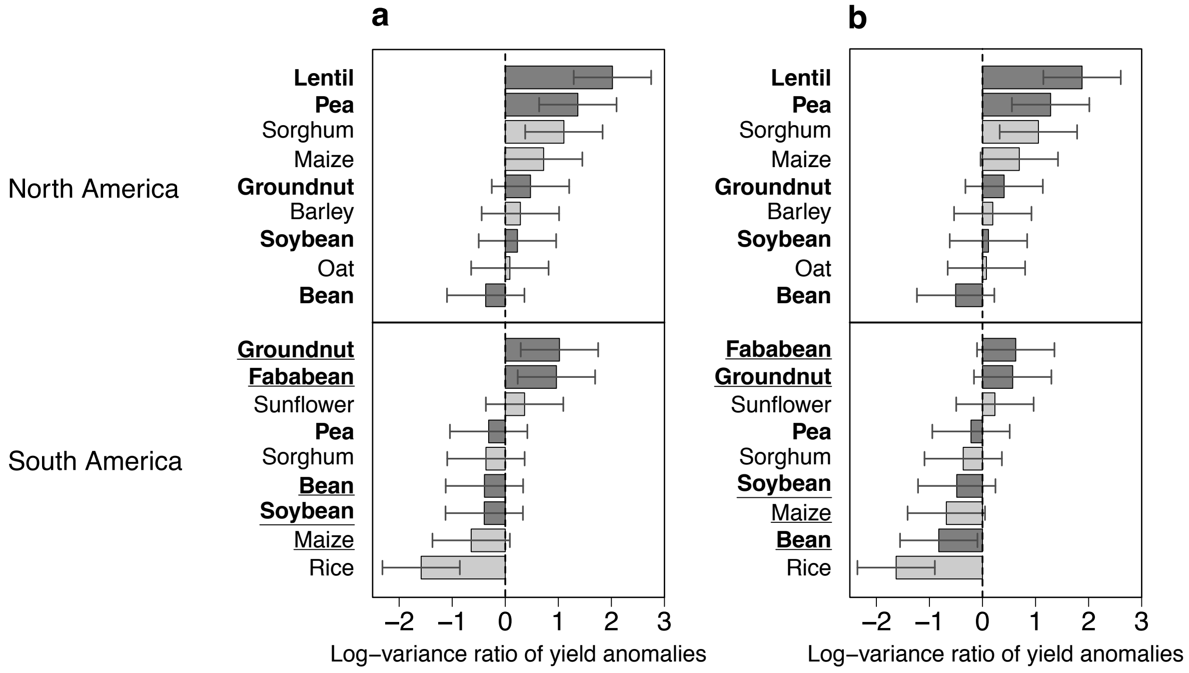
a
b
North America
South America
Lentil
Pea
Sorghum
Maize
Groundnut
Barley
Soybean
Oat
Bean
Groundnut
Fababean
Sunflower
Pea
Sorghum
Bean
Soybean
Maize
Rice
Fababean
Groundnut
Sunflower
Pea
Sorghum
Soybean
Maize
Bean
Rice
−2 −1 0 1 2 3
Log−variance ratio of yield anomalies

Supplementary Figure 8: Log-variance ratio of yield anomalies for 9 crops compared to wheat in two American regions over 1983-2013.

Variances are calculated over 1983-2013 and log-transformed. Yield anomalies are calculated using polynomial regression models (polynomial (a), loess (b)). Horizontal bars correspond to 95% confidence intervals. Among the 9 crops, 5 are legume crops (bold names and dark grey bars) and 4 are non-legume crops (non-bold names and light grey bars). All crops are ranked according to variance ratio values (decreasing order). Crops that change of ranking between polynomial and loess regression models used are underlined.

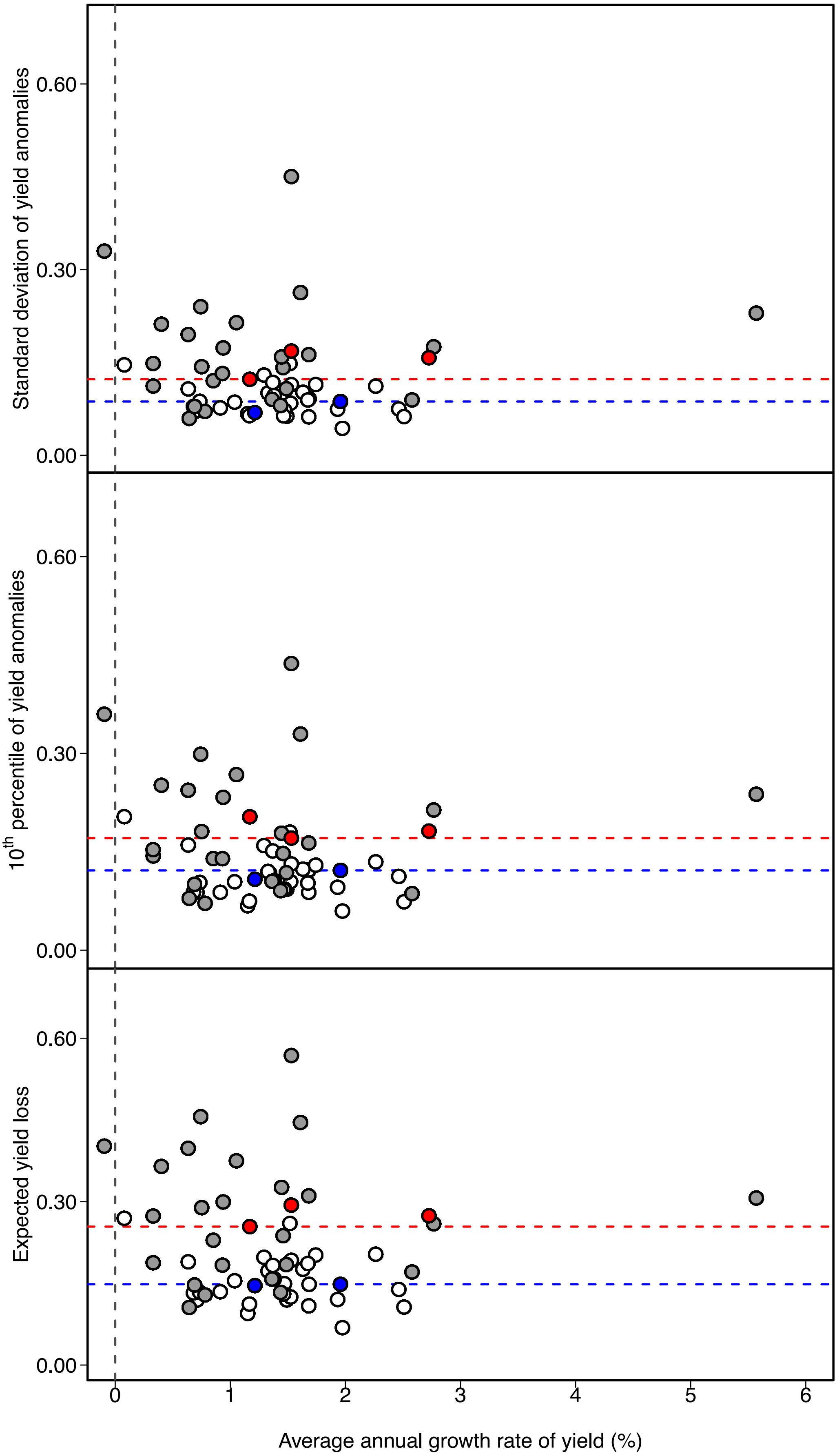

Standard deviation of yield anomalies
10th percentile of yield anomalies
Expected yield loss
0.00
0.30
0.60
0
1
2
3
4
5
6
Average annual growth rate of yield (%)

Supplementary Figure 9: Yield risk measures as a function of the average annual growth rate of yield in Europe and the Americas over 1961-2013 using the polynomial regression.

Risk measures are calculated over 1961-2013: standard deviation (a), 10th percentile (absolute values, b) and expected yield loss (absolute values, c) for all crop*region combinations. Crop*region combinations corresponding to legume and non-legume crops are indicated by grey points and empty points, respectively. The percentages reported in the x-axis correspond to average annual growth rate of yield (expressed in percentage) by a given crop in a given region over 1961-2013. Soybeans grown in the Americas and Europe are indicated in blue and red points. Blue dashed horizontal lines represent the maximal values of risk measures for soybean grown in the Americas. Red dashed horizontal lines represent the minimal values of risk measures for soybean grown in Europe. Grey vertical dashed lines represent the thresholds of 0% of average annual growth rate of yield. Yield risk measures and the average annual growth rate of yield are derived from yield anomalies calculated with the polynomial regression.

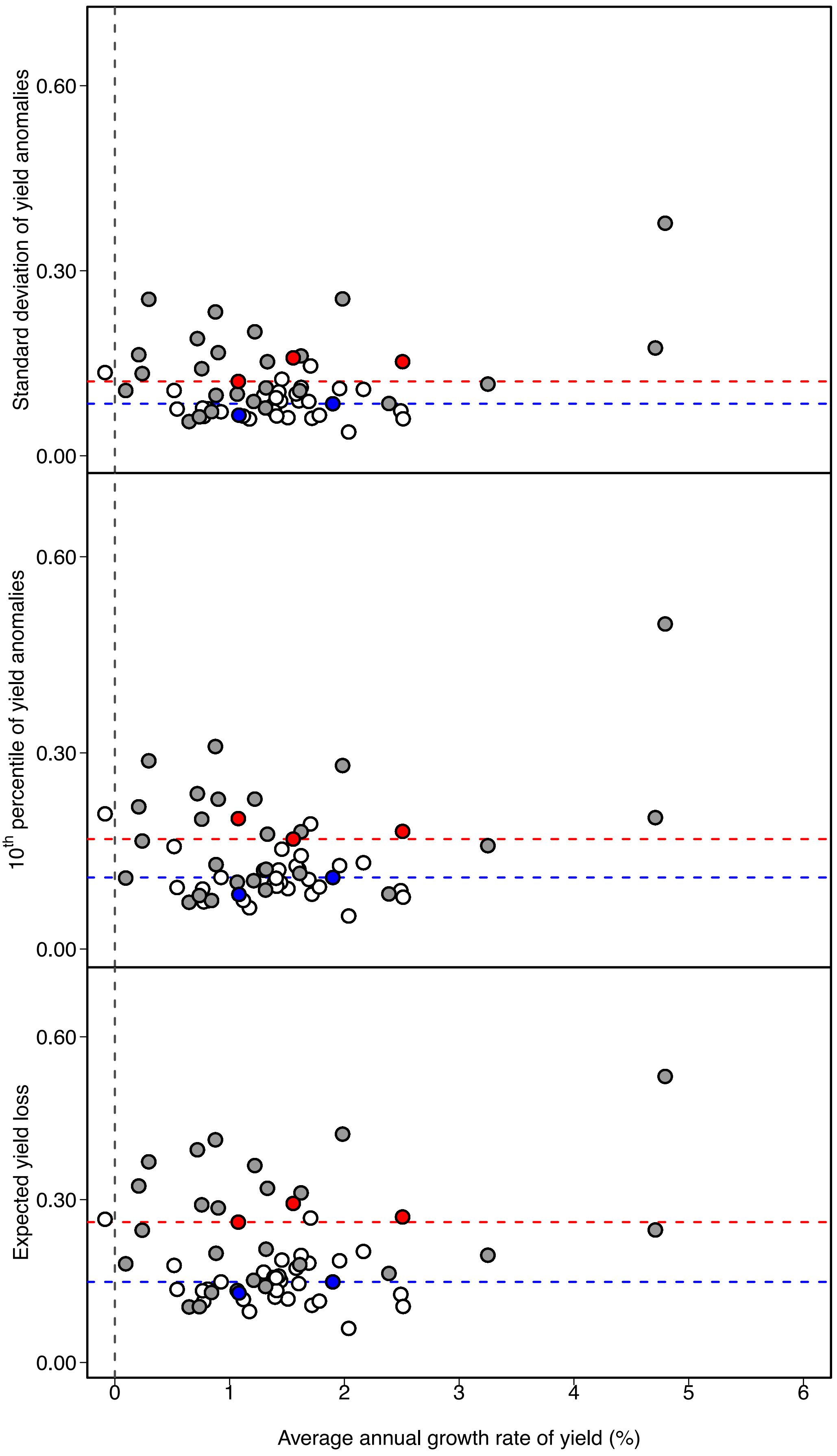

Standard deviation of yield anomalies
10th percentile of yield anomalies
Expected yield loss
0.00
0.30
0.60
0
1
2
3
4
5
6
Average annual growth rate of yield (%)

Supplementary Figure 10: Yield risk measures as a function of the average annual growth rate of yield in Europe and the Americas over 1961-2013 using the loess regression.

Risk measures are calculated over 1961-2013: standard deviation (a), 10th percentile (absolute values, b) and expected yield loss (absolute values, c) for all crop*region combinations. Crop*region combinations corresponding to legume and non-legume crops are indicated by grey points and empty points, respectively. The percentages reported in the x-axis correspond to average annual growth rate of yield (expressed in percentage) by a given crop in a given region over 1961-2013. Soybeans grown in the Americas and Europe are indicated in blue and red points. Blue dashed horizontal lines represent the maximal values of risk measures for soybean grown in the Americas. Red dashed horizontal lines represent the minimal values of risk measures for soybean grown in Europe. Grey vertical dashed lines represent the thresholds of 0% of average annual growth rate of yield. Yield risk measures and the average annual growth rate of yield are derived from yield anomalies calculated with the loess regression.

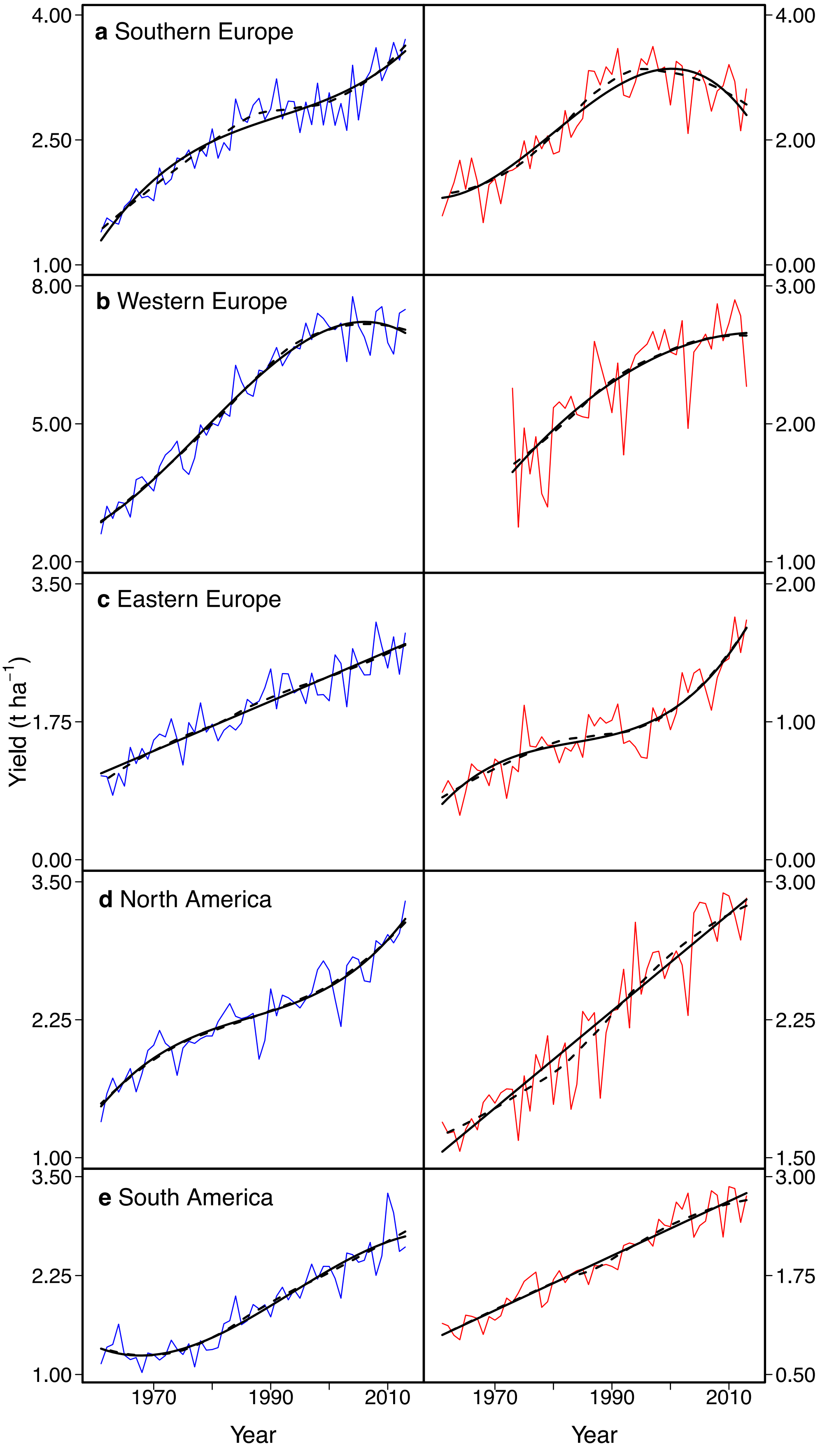
a Southern Europe
b Western Europe
c Eastern Europe
d North America
e South America
Yield (t ha^{-1})
Year
Year
1970
1990
2010
4.00
2.50
1.00
8.00
5.00
2.00
3.50
1.75
0.00
3.50
2.25
1.00
3.50
2.25
1.00
4.00
2.00
0.00
3.00
2.00
1.00
2.00
1.00
0.00
3.00
2.25
1.50
3.00
1.75
0.50

Supplementary Figure 11: Observed and fitted yield times series of wheat and soybean in Europe and the Americas over 1961-2013.

Observed yield time series for wheat (blue continuous line) and soybean (red continuous line) in three European regions and two American regions over 1961-2013. Polynomial (black continuous line) and local 'loess' (black dashed line) regressions models are fitted to yield time series. Northern Europe is excluded due to insufficient soybean yield data. Regions are ranked according to values of variance ratios of soybean yield anomalies compared to wheat (i.e., Southern Europe shows the highest soybean variance ratio compared to wheat).

Region	Country
Western Europe	Austria
	Belgium
	France
	Germany
	Luxembourg
	Netherlands
	Switzerland
Eastern Europe	Belarus
	Bulgaria
	Czech Republic
	Hungary
	Poland
	Republic of Moldova
	Romania
	Russian Federation
	Slovakia
	Ukraine
Northern Europe	Denmark
	Estonia
	Finland
	Iceland
	Ireland
	Latvia
	Lithuania
	Norway
	Sweden
	United Kingdom of Great Britain and Northern Ireland
Southern Europe	Albania
	Bosnia and Herzegovina
	Croatia
	Greece
	Italy
	Montenegro
	Portugal
	Serbia
	Slovenia
	Spain
	The former Yugoslav Republic of Macedonia
North America	Canada
	United States of America
South America	Argentina
	Bolivia (Plurinational State of)
	Brazil
	Chile
	Colombia
	Ecuador
	French Guiana
	Guyana
	Paraguay
	Peru
	Suriname
	Uruguay
	Venezuela (Bolivarian Republic of)

Supplementary Table 1: List of countries in the studied regions.

List of countries in the four European and two American studied regions. Countries are aggregated by the United Nations Statistics Division.

Region	Legumes				Non-legumes			
	Crop	Scientific name	*n*	Average cultivated area (10^6 ha)	Crop	Scientific name	*n*	Average cultivated area (10^6 ha)
Western Europe	Pea	*Pisum sativum*	*53*	0.32	Wheat	*Triticum* spp.	*53*	7.95
	Fababean	*Vicia faba*	*53*	0.07	Barley	*Hordeum* spp.	*53*	4.83
	Soybean	*Glycine max*	*41*	0.07	Maize	*Zea mays*	*53*	2.06
	Bean	*Phaseolus* spp.	*53*	0.02	Rapeseed	*Brassica napus*	*53*	1.44
	Lupin	*Lupinus* spp.	*53*	0.02	Rye	*Secale cereale*	*53*	1.34
Eastern Europe	Pea	*Pisum sativum*	*53*	3.40	Wheat	*Triticum* spp.	*53*	54.65
	Soybean	*Glycine max*	*53*	1.13	Barley	*Hordeum* spp.	*53*	24.84
	Vetch	*Vicia sativa*	*53*	0.72	Rye	*Secale cereale*	*53*	10.93
	Bean	*Phaseolus* spp.	*53*	0.67	Oat	*Avena* spp.	*53*	9.70
	Lupin	*Lupinus* spp.	*53*	0.35	Potato	*Solanum tuberosum*	*53*	8.86
Northern Europe	Pea	*Pisum sativum*	*53*	0.12	Barley	*Hordeum* spp.	*53*	4.37
	Fababean	*Vicia faba*	*53*	0.03	Wheat	*Triticum* spp.	*53*	2.85
	Vetch	*Vicia sativa*	*53*	0.01	Oat	*Avena* spp.	*53*	1.27
	Lupin	*Lupinus* spp.	*22*	0.00	Rapeseed	*Brassica napus*	*53*	0.67
	Bean	*Phaseolus* spp.	*53*	0.00	Potato	*Solanum tuberosum*	*53*	0.45
Southern Europe	Bean	*Phaseolus* spp.	*53*	0.59	Wheat	*Triticum* spp.	*53*	8.52
	Fababean	*Vicia faba*	*53*	0.28	Maize	*Zea mays*	*53*	4.13
	Soybean	*Glycine max*	*53*	0.23	Barley	*Hordeum* spp.	*53*	3.99
	Chickpea	*Cicer arietinum*	*53*	0.16	Oat	*Avena* spp.	*53*	1.05
	Vetch	*Vicia sativa*	*53*	0.13	Sunflower	*Helianthus annuus*	*53*	1.05
North America	Soybean	*Glycine max*	*53*	24.45	Wheat	*Triticum* spp.	*53*	33.80
	Bean	*Phaseolus* spp.	*53*	0.70	Maize	*Zea mays*	*53*	28.58
	Pea	*Pisum sativum*	*53*	0.61	Barley	*Hordeum* spp.	*53*	6.98
	Groundnut	*Arachis hypogaea*	*53*	0.59	Oat	*Avena* spp.	*53*	5.28
	Lentil	*Lens culinaris*	*53*	0.35	Sorghum	*Sorghum* spp.	*53*	4.33
South America	Soybean	*Glycine max*	*53*	17.56	Maize	*Zea mays*	*53*	17.24
	Bean	*Phaseolus* spp.	*53*	4.75	Wheat	*Triticum* spp.	*53*	8.56
	Groundnut	*Arachis hypogaea*	*53*	0.57	Rice	*Oryza* spp.	*53*	5.83
	Fababean	*Vicia faba*	*53*	0.17	Sunflower	*Helianthus annuus*	*53*	2.11
	Pea	*Pisum sativum*	*53*	0.13	Sorghum	*Sorghum* spp.	*53*	1.84

Supplementary Table 2: List of studied crops and their average cultivated areas over 1961-2013.

Five major legume and five major non-legume crops and their average cultivated area in four European and two American regions over the 1961-2013 period. Crops are ranked according to the values of average cultivated area (decreasing order). *n* is the number of years of yield data available for each crop in each region.
